# Value Co-creation in Non-profit Accommodation Platforms

**DOI:** 10.3389/fpsyg.2021.763211

**Published:** 2021-12-24

**Authors:** Vivian C. Medina-Hernandez, Berta Ferrer-Rosell, Estela Marine-Roig

**Affiliations:** Department of Business Administration, University of Lleida, Lleida, Spain

**Keywords:** non-profit sharing accommodation platforms, SD logic, value co-creation wellbeing, online travel reviews, qualitative research

## Abstract

Value co-creation, in the sharing accommodation sector, has been extensively analyzed but mainly with Airbnb as a reference and focusing mostly on guests’ perceptions. The aim of this study is to analyze the value co-created for users (guests and hosts) in the non-profit sharing accommodation platforms Couchsurfing and HomeExchange. This study also aims to analyze whether the co-created value of these platforms differs from that of for-profit platforms, along with how the outcomes, resources, and practices of the value co-creation process can help create wellbeing for individuals involved in the accommodation experience. Given that most of the existing literature on value co-creation in sharing accommodation platforms is based on Airbnb and guest perspectives, this study is a pioneer in analyzing how guests and hosts co-create value in the context of non-profit accommodation platforms using online travel reviews (OTRs) from non-profit platforms, and how the co-created value contributes to the wellbeing of the individuals involved. Results show that a set of tangible and intangible resources, such as the home and its amenities, helps users on non-profit platforms co-create value and that interaction and social practices between guests and hosts help co-create value for both groups. This implies that non-profit accommodation platforms may contribute more to the social dimensions of wellbeing of their users than for-profit platforms such as Airbnb where the host is typically absent from the experience. In addition, this study demonstrates that the co-created value in non-profit platforms depends on the *modus operandi* of each platform. On Couchsurfing, guests and hosts co-create more value from their social practices, and on HomeExchange, value co-creation depends more on tangible and intangible resources.

## Introduction

Technology and digitalization deeply impact the way people interact with each other. In the sharing economy, technology has helped to create new business models, such as Uber, Blablacar, and Airbnb. Each of these models operates using technology and, as Barbu et al. (2018) affirmed, the digital sharing economy has become important to society because it offers opportunities to generate revenue and increase social interaction that cannot otherwise be achieved (Barbu et al., 2018).

In the digital sharing economy, the accommodation sector has not been left behind. One of its most notorious and successful business models is Airbnb, the for-profit accommodation platform created in 2008 with millions of users or, specifically, hosts who list their accommodations on the platform, and travelers who use the application during their trips (Airbnb.com, 2020). Airbnb, thus, has attracted most of the attention of academics, and value co-creation is one of the subjects that has been the focus of their research (Camilleri and Neuhofer, 2017; Johnson and Neuhofer, 2017). Many studies have concluded that distinct value co-creation outcomes underlie certain resources and social practices (Camilleri and Neuhofer, 2017; Johnson and Neuhofer, 2017). Buhalis et al. (2020) expanded on this, analyzing not only value co-creation but also co-destruction and how the use of these platforms can affect the wellbeing of individuals and the community.

Ryan and Deci (2001) described wellbeing as a construction that involves optimal experience and functioning. Wellbeing is then perceived as a subjective evaluation of life in terms of experiences, positive feelings, and satisfaction (Seligman, 2002) that is constructed or co-created in community. The use of this kind of digital sharing model might affect not only individuals but also community wellbeing (Buhalis et al., 2020). However, the study of Airbnb as a representative model of a sharing economy has also been criticized due to its capitalist practices and for-profit model. Some researchers have called Airbnb an “on-demand” economy model (Cockayne, 2016) rather than collaborative consumption or a collaborative economy. The sense of community as a sharing economy goal is difficult to evaluate in this platform, not only because the host is mostly absent during the guest experience but also because individuals are transformed into commercial partners rather than belonging to a social community.

Until now, value co-creation and the co-creation of wellbeing in the touristic sharing economy has been studied mostly on Airbnb (Breidbach and Brodie, 2017; Camilleri and Neuhofer, 2017) and not on non-profit platforms, even though these platforms may be more aligned with the concept of the sharing economy where users have different profiles and motivations (Medina-Hernandez et al., 2020). Thus, the value co-created between users and the possible wellbeing obtained in the value co-creation process on non-profit platforms may be different or even superior.

This study analyzes the value co-creation between guests and hosts under the consideration of the theoretical framework of service-dominant (SD) logic introduced by Vargo and Lusch (2004) and the theoretical framework of value co-creation in the Airbnb context exposed by Johnson and Neuhofer (2017). We adapted these frameworks to non-profit accommodation platforms and analyzed how this possible value might affect the wellbeing of their users. SD logic suggests that value is created not only by firms but also by customers who integrate operand and operant resources to extract value. Johnson and Neuhofer (2017) used SD logic in the context of Airbnb to analyze how guests co-create value through resources, practices, and outcomes. However, they merely considered guest perspectives in the value co-creation process, leaving out the possible contribution of the host when creating value during the accommodation experience. That might be because, in many cases, Airbnb hosts are absent during the guest accommodation experience (Martin, 2016; Murillo et al., 2017; Mahadevan, 2018). However, in some non-profit platforms, such as HomeExchange or Couchsurfing, the host plays a significant role during the stay and sometimes even before and/or after.

In this respect, some authors have argued that the value obtained from sharing platforms is not re-invested in the local community (Ozanne and Ozanne, 2016). Many believe that sharing platforms promote the opposite of wellbeing for their users by creating a new subclass of low-income earners with little security (Guerrini, 2015). However, this paradigm seems more suitable to a for-profit platform scenario. A limited amount of research has been conducted to investigate the impact of the sharing economy from the perspective of residents. For example, in their analysis of residents’ risk perception of P2P accommodation, Camprubí and Garau-Vadell (2021) identified that residents’ risk perception of P2P accommodation is not standardized and that there are levels of risk perception. By contrast, other researchers have stated that sharing-economy markets help their users feel that they are part of a community and, thereby, promote pro-social values (Ozanne and Ozanne, 2016). For instance, Batle et al. (2020) analyzed the motivations of locals for meeting tourists in the context of experience exchange in P2P accommodation. They found that a passion for the sharing accommodation activity and personal attributes, such as good communication and interpersonal skills, are major motivational factors for locals to get involved in the P2P accommodation experience.

The current study aims to investigate whether non-profit sharing accommodation platforms are helping to create a sense of community and pro-social values and contributing to the users’ individual wellbeing, given the host-guest interaction is habitual in this kind of platform. The analysis scope is Barcelona. It was considered due to its importance as a tourist destination and the impact and controversy sharing accommodation platforms have generated in the city in recent years (Huertas et al., 2021). In 2018, the city ranked 17th in the world on the list of most visited cities by international tourists and 8th among European urban destinations (Statista.com, 2021).

## Literature Review

### Service-Dominant Logic and Peer-to-Peer (P2P) Sharing Accommodation

Service-dominant (SD) logic by Vargo and Lusch (2004) is perceived as a general change in the perspective of the production process, where the consumer is involved as a co-producer and recognizes his/her involvement as necessary to “fit his or her needs” (Vargo and Lusch, 2004, p. 35). In other words, value is not created “pre-consumption” or given to a product or service by its producer but only through the consumption process. In this process, certain resources intervene: “operant resources,” or intangible elements such as knowledge and human skills, and “operand resources,” or tangible elements such as amenities, equipment, and physical products that must be activated by operant resources to make them valuable (Vargo and Lusch, 2004; Johnson and Neuhofer, 2017).

The SD logic theoretical framework has been adopted by researchers in many fields, including marketing and business (Quero and Ventura, 2019), technology (West et al., 2018; Neuhofer et al., 2020), and healthcare (Chakraborty et al., 2014). In the accommodation context, tourists perceive accommodation as an integrating element of the whole travel experience (Költringer and Dickinger, 2015; Lalicic et al., 2021). New experiences are one of the motivational elements that both guests and hosts look for when they sign up for sharing accommodation platforms (Guttentag, 2015; Tussyadiah, 2016). To create experience and value, guests and hosts must interact and integrate operant and operand resources (Johnson and Neuhofer, 2017).

Johnson and Neuhofer (2017) used the conceptual framework of SD logic to analyze value co-creation through online content reviews of Airbnb guests in Jamaica. Their study illustrates the resources and value outcomes of Airbnb users embedded in the authentic culture of the destination. In addition, Buhalis et al. (2020) used SD logic to explore value co-creation and co-destruction of the accommodation sharing economy and investigate the role of individual stakeholders. However, concerns about “absent hosts” or a lack of sharing practices have risen in paying platforms such as Airbnb (Murillo et al., 2017; Dolnicar, 2019; Ert and Fleischer, 2019), which could greatly limit value co-creation during the experience. In this respect, the SD logic theoretical framework has not been extrapolated to the non-profit sharing accommodation platform scenario, where the host-guest interaction is more intense. Thus, it is highly interesting to analyze how the guests and hosts of this kind of model co-create value, which operant and operand resources act in the value co-creation process, and, ultimately, how this may contribute to the individuals’ wellbeing.

### Value Co-creation in Airbnb

Value co-creation practices in Airbnb as a model of the sharing economy have been analyzed by several researchers. Sthapit et al. (2019) studied the co-creation practices obtained from the use of Airbnb. They applied a web survey to a sample of 259 people. Their results showed that co-creation and non-information overload helped user satisfaction. Casais et al. (2020) conducted 30 in-depth interviews with Portuguese Airbnb hosts. Their results showed that the hosts built a close relationship with their guests, which was crucial for the co-creation of the tourism experience.

On the other hand, using the SD logic as a theoretical framework, Jiang et al. (2019) used a two-stage survey to examine the co-creation of customer-perceived value between Airbnb hosts and guests. Results from their studies showed that Airbnb value facilitation and host value facilitation affect economic value, emotional value, green value, and ethical value.

Camilleri and Neuhofer (2017) analyzed value co-creation and co-destruction from guest and host social practices in the context of Airbnb through a quantitative content analysis from reviews posted by users in Malta. Their findings showed how certain guest-host social practices resulted in a range of value formation dimensions. Thaichon et al. (2020) analyzed the factors that lead to satisfaction and value co-creation in the Airbnb sharing economy context. They used semi-structured interviews applied to Australian guests and hosts. Their analysis resulted in six primary factors and three secondary factors that affected both guest and host satisfaction and value co-creation.

Most of the research on Airbnb value co-creation has used qualitative content analyses through the conduction of structured or semi-structured interviews as a way of collecting data. The analyses have been geographically centered, with most of them in destinations with a large presence of Airbnb listings. However, the study of how the interaction between guests and hosts may affect individual wellbeing and the destination itself has scarcely been mentioned.

### Value Co-creation in HomeExchange and Couchsurfing

HomeExchange is a non-profit accommodation platform where users exchange their homes without exchanging money. The exchange can be simultaneous or not. Although there is no money exchanged between hosts and guests, users must pay a fee to register on the application (HomeExchange.com, 2021). Couchsurfing is another non-profit accommodation platform that does not require monetary exchange between its users. Guests book a “couch” in their host’s accommodation (although it can be a room). Before the COVID-19 pandemic, the use of the platform was free. Nowadays, users must pay a small fee to use the service (Couchsurfing.com, 2020).

Literature on value co-creation in non-profit accommodation platforms is limited. Sevisari and Reichenberger (2020) used value co-creation to analyze hosting experiences on the Couchsurfing non-profit platform in Indonesia by applying 20 in-depth interviews and a focus group. Results showed the value practices and the value outcomes of the host experiences, which were highly related to friendship, cultural knowledge, and self-employment opportunities. Regarding literature on value co-creation in HomeExchange, there were no findings on this topic.

Appreciating local resources and respecting the needs of hosts and guests can generate new, interesting, and even fulfilling experiences through value co-creation (Buhalis et al., 2020). This panorama has been studied in the context of Airbnb, but until now, there has been no research on how the resources and outcomes of hosts and guests on non-profit platforms can co-create value and generate wellbeing for its users and for the destination’s community.

### Wellbeing Within the Framework of Value Co-creation

According to Ryan and Deci (2001), the concept of wellbeing implies an optimal experience and psychological functioning, where trusting and supportive interpersonal relationships have fundamental importance. There is also the concept of community wellbeing, which is described as “the satisfaction with the local place of residence considering the attachment to it, the social and physical environment, and the services and facilities” (Forjaz et al., 2011, p. 734). Another description of community wellbeing is related to a state that facilitates the pursuit of individual and group goals (Armitage et al., 2012).

**FIGURE 1:**
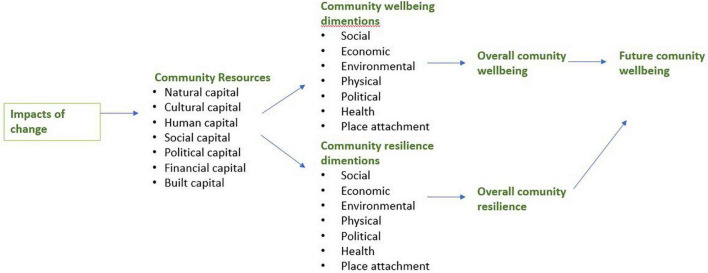
A conceptual framework for community wellbeing and resilience by Mccrea et al. (2016).

In their research regarding community wellbeing and resilience, Mccrea et al. (2016) proposed a conceptual framework for both, stating that the relationship between wellbeing and resilience becomes stronger when there is a challenge or a rapid change facing a community (see Figure 1).

An impact on community resources affects community wellbeing and resilience dimensions. However, the relationship between resources and dimensions is not lineal but more complex. “Just because a community has many resources, it does not necessarily mean that they will be mobilized in response to changes in community wellbeing, especially if these changes are gradual or if important resources are privately controlled by [a] small minority” (Mccrea et al., 2016. p 197).

Individuals perceive happiness and satisfaction subjectively but generally co-create it socially (Buhalis et al., 2020). In the context of the sharing accommodation sector, this implicates not only local hosts but also their neighborhoods, communities, and resources (Luhmann, 1995; Zhang and Veenhoven, 2008).

The construction of wellbeing has been analyzed from several perspectives. Ryan and Deci (2001) reviewed the antecedents of wellbeing, or the conditions that facilitate it. In their study, they organized a review of three subjects: wealth, relationships, and goal pursuits. Regarding relationships as an antecedent of subjective wellbeing, research on the quality of relationships has found that individuals who “in general, have more intimate or higher-quality relationships tend to demonstrate greater wellbeing” (Ryan and Deci, 2001, p. 51). Research by Reis et al. (2000) showed the type of interactions that promote wellbeing. They argued that when people “felt understood, engaged in meaningful dialog, or had fun with others,” their wellbeing was higher.

Regarding the social variable of individual wellbeing, Ryff et al. (2001) found that positive relations had an impact on some physiological functioning, such as the secretion of oxytocin, which is associated with stress relief and a positive mood.

Using the categorization scheme of Johnson and Neuhofer (2017) and based on the theoretical framework of SD logic, this study attempts to analyze: (1) how the possible value co-created between guests and hosts through operand and operant resources, social practices inherent to the improvement of wellbeing, and co-created outcomes can contribute to the wellbeing of both kinds of users; and (2) if those results are different in each non-profit sharing accommodation model.

## Materials and Methods

### Barcelona as a Case of Study

Barcelona is one of the most visited cities worldwide (Euromonitor International, 2020), attracting the high demand of tourists (in a pre-pandemic context). Barcelona attracts all kinds of tourist profiles, from entire families to solo travelers looking for adventure. The city provides all kinds of accommodation, from traditional hotels, hostels, and “on-demand” economy models listings (Airbnb) to non-profit accommodation listings (HomeExchange and Couchsurfing). At the moment of writing this paper (June 2021), there were more than 17,500 Airbnb listings, 13,000 HomeExchange listings, and 23,000 Couchsurfing listings in Barcelona. It is well known that Barcelona has affronted many concerns by local people regarding the rising number of tourists in the city. From 2018 to 2019, the number of homes for tourist use and beds increased by 8.7 and 8.2%, respectively, in the city (Barcelona City Hall, 2021).

This situation has affected the lives of locals, and research has been done regarding the matter. In this respect, Huertas et al. (2021) analyzed the treatment of Airbnb controversial issues in the Spanish press and their evolution. Their analysis revealed the term “Barcelona” was frequently accompanied by terms with a negative connotation (e.g., “gentrification”) that referred to the Airbnb phenomenon negatively affecting the destination.

Due to the prominence of the Airbnb phenomenon, Barcelona has been a case study in the context of sharing accommodation in several studies. Gutiérrez et al. (2017) analyzed the spatial patterns of Airbnb in Barcelona and compared them with hotels and sightseeing spots. They highlighted that Airbnb capitalizes more on the proximity to main tourist attractions in comparison with traditional hotels and also detected the spots with more tourist pressure due to the presence of Airbnb listings. Garcia-Ayllon (2018) analyzed the effect of “tourist flats” on the Airbnb platform in three Spanish cities, including Barcelona. Their results showed “worrying indicators” regarding the economic and social sustainability of the city’s tourist model. In addition, Lladós-Masllorens et al. (2020) studied the pricing strategy of listings in Airbnb in Barcelona and found that a systematic interaction of valence and volume of online reviews can produce a crucial impact on the listings’ pricing. Other authors have found, through Airbnb reviews in Barcelona, that the location of the accommodation strongly influences the online travel review (OTR) narrative, which may affect the destination’s image, tourist satisfaction, and loyalty (Marine-Roig, 2021).

In this context, Buhalis et al. (2020) made a critical analysis of the situation of the sharing accommodation listings in Barcelona and their impact on community wellbeing. However, although Barcelona has been intensely studied in relation to Airbnb and its (mostly negative) impacts, we have yet to see what the role of non-profit accommodation platforms is in the city in relation to value for its users and wellbeing.

### Methodology

**FIGURE 2:**
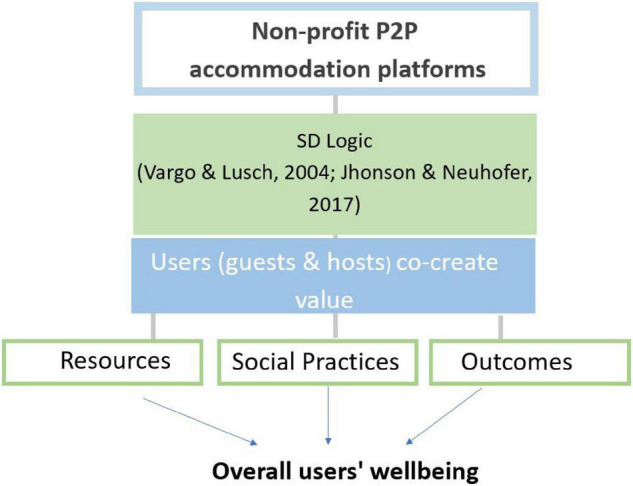
Proposed theoretical framework of wellbeing in value co-creation in non-profit P2P accommodation platforms.

The study aims to analyze and explore the possible value co-creation resources, social practices, and outcomes in non-profit sharing accommodation platforms using the SD logic theory as a conceptual framework (Vargo and Lusch, 2004) and the theoretical framework from Johnson and Neuhofer (2017). It also seeks to analyze how this co-created value might affect the well-being of users (see Figure 2). This study conducted a qualitative analysis through a random sample of 900 OTRs from guests and hosts of the non-profit sharing accommodation platforms HomeExchange and Couchsurfing (450 reviews each), published in 2019, from the city of Barcelona. As Corbin and Strauss (2014) stated, qualitative methodology is useful to analyze the experiences of participants and their thinking and reflective processes, and to identify their relationship between their previous knowledge about the destination and their culture. This study analyzes online travel reviews (OTRs), which provide information regarding the post-purchase experiences of customers. If such reviews are analyzed efficiently, they are valuable for obtaining high-quality customer experience insights (Guo et al., 2019). Qualitative methods have been used to analyze OTRs due to the improbability of decontextualizing the experiences of participants (Guo et al., 2019). Tussyadiah and Zach (2017), in their quantitative study regarding attributes of P2P accommodation experience, identified not having applied qualitative methodology as a limitation, leading to concerns that valuable information may have been lost in the data, and that a qualitative analysis of selected samples of reviews may be useful for ensuring the precision of big data analysis. The present study applies qualitative methodology on OTRs given the experiential basis of that type of data. A literature review confirms that other studies have also implemented qualitative methodology for the analysis of user-generated content (UGC), such as the work by Bigne et al. (2020) that examined memorable tourist experiences and implemented a qualitative analysis of 8,074 online reviews. Additionally, Camilleri and Neuhofer (2017) conducted a quantitative online content analysis of 850 guest reviews on Airbnb. Outside the tourism context, Welhausen and Bivens (2021) explored UGC for a civilian mobile first health responder using qualitative methodology.

For data collection, a total of 12,336 reviews from non-profit platforms published during 2019 were extracted using the web scraper software Webharvy version 6.0.1.173. The manual data refining considered only those reviews in the English language. From this first filter, a total of 2,657 reviews remained. Reviews from HomeExchange included reciprocal and non-reciprocal exchanges. Finally, a cleaning process was conducted on the 2,657 reviews considering the following criteria: listings with less than 10 reviews were eliminated, along with reviews with automated responses; cancelations were not considered; and only reviews with at least five words were selected. From this cleaning process, 900 reviews were manually and randomly obtained. The representativeness of the sample in this study can also be argued by the reading process, which means that once the data source no longer generates new information for the particular case of study, the data acquisition process can be stopped, and no more OTRs need to be read (Lewis et al., 2003).

While 900 OTRs from the Airbnb platform were also analyzed, results were merely used to compare and discuss the results obtained from the data analysis of the non-profit platforms due to extension limits. Reviews from Airbnb included both private and shared rooms. The extracted data were subsequently imported, organized, and analyzed with the ATLAS.ti Software Version 9 (Rambaree, 2014). Based on the qualitative content analysis methodology (Erlingsson and Brysiewicz, 2017), with the support of the ATLAS.ti software, the coding structure was constructed by following the framework used by Johnson and Neuhofer (2017) and adding the hosts’ reviews to the analysis. Studies addressing for-profit sharing accommodation platforms attempt to determine what values, if any, apart from the monetary benefit are co-created by the hosts (Böcker and Meelen, 2017; Tussyadiah and Park, 2018; Oskam et al., 2018; So et al., 2018). In this study, we analyze the perspectives of hosts beyond the monetary motivation, considering how the platforms operate. The social benefit, which is more aligned to the sharing economy discourse, and its link to subjective wellbeing (DeNeve, 1999) makes the hosts an important element to analyze in the value co-creation process.

**FIGURE 3:**
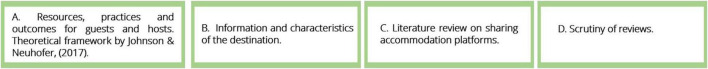
The process of creating the coding structure.

In terms of data categorization and analysis, this study applies a grounded theory design. A grounded theory approach involves the application of inductive reasoning to develop a theory through the collection and analysis of participants’ data (Strauss and Corbin, 1994; Charmaz and Smith, 2003). This study adopts five steps for a grounded theory approach in the coding structure as can be seen in Figure 3:

(A) This study departs from inductive reasoning, working with pre-applied categorization coding (Johnson and Neuhofer, 2017) but adding and modifying the subcategories resulting from the analysis, and involving the host in the analysis, which implies deductive reasoning. Such qualitative analysis cannot be strictly codified; therefore, it also requires a degree of intuitiveness regarding what the data mean (Thaichon et al., 2020). (B) This inductive-deductive methodological process of qualitative content categorization also considered the information and characteristics inherent to the destination that might influence the value co/creation process. (C) The reviewed literature on for-profit and non-profit sharing accommodation platforms, and (D) the scrutiny of the 900 OTRs collected.

In some cases, a review was categorized into different categories because it had information related to different aspects.

## Results

Drawing from the SD logic theory by Vargo and Lusch (2004) and applying the codification used by Johnson and Neuhofer (2017), Tables 1, 2 show the resources, practices, and outcomes involved in the value co-creation process in the non-profit sharing accommodation platforms Couchsurfing and HomeExchange.

**TABLE 1:** Value co-creation of hosts and guests in Couchsurfing.

4.1 Hosts and guests’ value co-creation in Couchsurfing					
4.1.1 Value co-creation resources			4.1.2 Value co-creation social practices		4.1.3 Value co-creation outcomes

4.1.1.A Guest resources		4.1.1.B Host resources	Guest	Host	
**Operand resources**		**Operand resources**	**Touring and discovering** **the destination together**	**Touring and discovering** **the destination together**	
***Home***	***Places***		-Discover Barcelona by bike	-Go for a walk and visit cultural places	
-Clean	-Beach		-Go to social events (parties, concerts)	-Going out for a drink and party with personal friends	
-Cozy	-Near villages		-Do relaxing plans together (watch the sunset at the beach, picnics)		**Construction of a friendship based on trust**
-Well located	-Parks		-Visit emblematic neighborhoods		
	-Attraction points		-Go to local restaurants and bars		**Intention of repeating the encounter and recommendation**
	-Bars and restaurants				
	-Discos		**Cultural learning** **from host**	**Cultural learning** **from guest**	Experience the Couchsurfing “spirit”
			-Have interesting conversation and discussions	-Eating local food prepared/gifted by guests	
**Operant resources**		**Operant resources**	-Practice the local language	-Discover new places trough guest’s stories	
***Host***		***Guest***	-Learn how to prepare authentic local dishes	-Learn how to dance new rhythms	
-Helpful		-Talkative			
-Friendly		-Clean	**“Engage” with host trough activities**	**“Engage” with guest trough activities**	
-Multilingual		-Tidy	-Have breakfast, lunch, dinner with the host	-Enjoy and learn from guest’s passions, jobs	
-Excellent guide		-Respectful	-Enjoy host’s free time	-Do leisure activities (go to the cinema, practice yoga, go to a spa)	
-Flexible		-Friendly	-Practicing sport with host		
-Funny		-Trustworthy	-Play board games		
-Interesting		-Independent			
-Easy to talk		-Sociable			
-Good cooker					

**TABLE 2:** Value co-creation of hosts and guests in HomeExchange.

4.2 Hosts and guests’ value co-creation in HomeExchange						

4.2.1 Value co-creation resources				4.2.2 Value co-creation social practices		4.2.3 Value co-creation outcomes

4.2.1.A Guest		4.2.1.B Host		4.2.2.A Guest	4.2.2.B Host	
**Operand resources**		**Operand resources**		**Enjoying the home as their own**	**Communicating with guest**	
***Home***	***Places***			-Having a drink/meal from the terrace	-Exchanging photos and information	
-Kid Friendly	-Beach			-Enjoying the view from the balcony/rooftop		
-Location	-Metro station			-Relax at the pool/garden after long days sightseeing		**Desire of repetition**
-Amenities	-Neighborhood			-Kids playing with the toys left for them		
-Clean	-Shops			-Taking care of animals and plants		**Users’ recommendation**
-Quiet	-Supermarkets					
	-Bars and restaurants					**Construction of a long-term relationship**
	-Tourist attractions					
	-Kids ‘park			**Exchanging information with host**	**Doing activities with the guest (before, after or during)**	
**Operant resources**		**Operant resources**		-Learning about cultural and historical information	-Dinner together	
***Host***	***Communication***	***Guest***	***How gests left the home***	-Host’s personal life	-Sightseeing	
-Informative	-Clear	-Nice	-Organized	-Recommendations	-Exchanging gifts	
-Helpful	-Constant	-Good language skills	-Clean		-Kids playing together	
-Friendly	-Efficient	-Respectful	-Just as they found it	**Feeling like a local**		
			-Found it better	-Eating in local bars and restaurants		
				-Shopping in the neighborhood		
				-Use the car and visiting near villages		
				-Enjoying not crowded places		
						

### Value Co-creation in Couchsurfing

#### Value Co-creation Resources

##### Guest’s Resources

Vargo and Lusch (2016) argued that, in the consumption process, consumers co-create value integrating operand, or tangible, resources and operant, or non-physical, resources. In the context of the tourism sector, an example of operand resources would be places of interest, while operant resources might be exemplified by the time used to visit said attractions (Jiang et al., 2019). In the Couchsurfing sharing economy model, results show the presence of three major resources within the guests’ value co-creation process, as observed in Table 1.

###### Home

Although the accommodation is not what most guests care about during their experience, it seems that some aspects are relevant as an operand resource. Reviews revealed a service dominant need for a *clean* home.

- *“He was very welcoming; his flat is very clean and the couch very comfortable”.*
- *“Christine and Kevin are so kind and friendly. Their house is clean.” “Very nice and clean apartment.”*
- *“His apartment is cozy and clean. I slept in his bed while he moved to the couch.”*

Guests do not typically spend much time there, thus they do not ask for a lot of resources and have minimal standards. Couchsurfing guests, instead, indicate their need to stay in a *clean, well-located*, and *cozy* home without asking or expressing their needs for extra amenities.

- *“Location of his flat is the best for exploring the city and I love design of his flat.*
- *“It is really unusual.” It is located next to historical places and has a very modern design.”*
- *“which by the way is conveniently located in a little east side near the city center.”*

###### Places

Reviews suggested that some places are relevant operand resources that are integrated in the guests’ value co-creation process, but they are always activated by the presence of the hosts. The same places would not create value without the hosts’ integration action. Visiting those places with the host helped guests to experience them from a non-tourist perspective (the perspective of someone who lives there and has local knowledge of those places).

- *“They showed us amazing places in Barcelona, we were going for a ride in the car, visited beach and wonderful place in the hills with beautiful view of the night city. Thank you, guys. We appreciate your help!”*

In this case, the most visited places were the beach, where hosts and guests did diverse activities together; local bars and restaurants, where guests enjoyed typical food and drinks and had fun; parks with views of the city; and small towns near Barcelona, where guests had the opportunity to experience a more authentic tourist experience.

- *“Super knowledgeable about local culture and history and customs especially at the town festival in Sitges where we ended up going and danced to a lot of Catalan songs in the night and saw human towers in the morning.”*
- *“I think of how we discovered bcn, tried new veggie locations and restaurant, did a picknick in a gorgeous park, enjoyed a water light concert and time in cool life music bars, we went to a concert and that we dance and club or chilled at the beach with sunset*…*We did a lot of things and I enjoyed everything!”*

Reviews also revealed that places that co-created value in the guest experience were generally not the most touristic places.

###### Host

For Couchsurfing guests, the host is the operant resource that helps to integrate other resources and becomes fundamental in the value co-creation process. For example, when the host becomes a guide in the destination, he/she integrates a place during the guest’s value co-creation process, making that place more special or useful.

Reviews show that hosts attempted to co-create value when they were *helpful, friendly, flexible*, or *funny.*

- *“Gentle, helpful, friendly, very funny and attentive, we had (my friend Heloise and I) a very good time with him!”*
- *“I got in late to the airport and he was in the area and offered to pick me up which was so awesome!”*

Some of these aspects might also appear in the context of a for-profit platform, but there are others that clearly denote the guest-host interaction, which describe new characteristics of the host to facilitate value, such as *easy to talk to, multilingual, good cooker*, or *excellent guide.*

- *“He really funny and really good cooker (as a lot of people already said).”*
- *“He even cooked me some Spanish dish, which was SOOOOO good, and got muffins.”*
- *“He is a good guide in the city!!! I definitely recommend him.”*

##### Resources of Hosts

Contrary to guests on Couchsurfing, during the hosts’ experience, value emerges more through social practices than resources. Reviews showed that the *guest* was the operant resource that contributed to value co-creation in the hosts’ experience.

###### Guest

A guest is a necessary operant resource for value to emerge in the hosts’ experience. Although places are highlighted in the host reviews, none of them would generate value for the host in the context of Couchsurfing without the presence of the guest. This can be argued since the host lives in the destination and knows those places, but it is the tour with the guests around those places that makes the value emerge. However, clearly, this value is not co-created by the guests themselves. For value to be co-created, guests must express characteristics such as talkative, clean, tidy, or respectful.

- *“Inés is very easy-going, talkative and incredibly warmhearted.”*
- *“She is clean, easy-going girl and respectful person.”*
- *“He was very clean and tidy too, and also very quiet while I was sleeping.”*

Other characteristics, such as *trustworthy*, are highlighted, and building trust is one of the characteristics in the Couchsurfing model. Molz (2013) coined the term “moral affordability,” which explains how trusting strangers share physical assets and form successful social relationships.

- *“I think that Katja is a person in which I could trust, I could have hosted her more days*…”
- *“I know that is girl at who you can trust! I can host her again anytime.”*

This is precisely what the model of this platform is about. In a for-profit model, such as Airbnb, the host has somehow insured his possessions, since the expenses are charged to the guest’s credit card through the platform. However, on Couchsurfing, guests and hosts must learn to trust each other to experience enriching social practices. In addition, to co-create value, guests must be *independent*, *openminded*, and *sociable.* Most hosts expressed that it was important for them that their guests got along well with their personal friends.

- *“She got along with all my friends and fit so well in all activities we had together.”*

#### Value Co-creation Practices

Reviews showed that social practices in this platform are relevant to co-create value. As the analyzed reviews indicated, the social practices between both users within a local environment contributed to the appearance of value. Subcategories related to social practices are considered the same for both guests and hosts, although practices or activities that created value resulted differently for each one.

##### Touring and Discovering the Destination Together

Guests expressed a desire to share time with their hosts. For them, it was valuable that someone who lives in the destination could give them a tour, explain historical and cultural facts, and enjoy typical activities.

- “*Spent nice time at home and he showed me some places in city with his bike. It was amazing.”*
- *“Alex is a really funny and friendly guy. He cared to make me discover lot of interesting and various spots of Barcelona. We went discovering Barcelona together, great places, typical Catalan food experience, we also partied, and they really made me discover wonderful things from their life and from the European culture.”*

It was also valuable for them to be involved in the personal activities of the hosts, for example, going to parties with the hosts and their friends or accompanying them to activities after work.

- *“He kindly invited me to the birthday party for one of his friend’s, which was held on day I arrived in Barcelona. I had such a great time there (even though I cannot speak a word of Spanish)!”*
- *“She invited me to this international event organized by his co-workers, and I had a great time there.”*

Hosts, on the other hand, enjoy showing the city and their favorite places and teaching the cultural or historical background of the destination. They enjoyed the possibility of “rediscovering” the city by showing it.

- *“We took them to our favorite spot in the city. Thanks for the unforgettable micheladas.”*
- *“We visited the city together and we went to the wonderful party Churros con Chocolate. We went to grab beers but everything ending in a party with another funny team.”*

In this practice, value emerges from the interaction between guests and hosts, but it can also involve other local people, making the social interaction more enriching for both users. These practices involve the local community and spaces outside of the accommodation.

##### Cultural Learning

Cultural learning is one of the relevant motivations for Couchsurfing users. Aydin and Cihan Duyan (2019) pointed out that users (hosts and guests) join this platform for several reasons, such as obtaining knowledge about other cultures. This can be difficult to achieve in the mass-tourism era, where tourists, places, and experiences are “commodified,” and the cultural contribution of a destination is “dramatized” for economic purposes. Results showed that guests and hosts learned about each other’s culture mostly in activities done inside the accommodation.

Guests enjoyed having conversations and discussions with hosts, from which they obtained cultural learning. Conversations also helped them get to know each other better and establish deeper connections.

- “*We had good conversations about our lives ☺He is also very responsive in WhatsApp. I also appreciated our conversations a lot.”*
- *He always raises some interesting questions which make me think and realize more about how cultural differences shape different thoughts.*
- *“We learnt a lot sharing experiences.”*

In addition, practicing the local language was an activity through which value could be acquired. Guests value being able to practice the local language with the host, but also, having a host who speaks their native language is also valuable, thus enriching the cultural exchange.

- *“We‘re very happy that could spend some time together and get some Spanish classes.”*
- *“He’s one of the most interesting people I’ve ever met, we could’ve talk for hours and change between Spanish, French, English; he also speaks Portuguese, Catalan and he’s learning Russian, German and mandarin and knows so many about other languages and countries.”*

Learning how to prepare typical meals was an activity that helped value appear. Reviews showed that some guests learned how to prepare typical food of the destination from their hosts, which was also important to get involved with the local culture and strengthen their relationship with each other.

- *“He is an exceptional cook who taught us how to prepare paella!”*
- *“We stayed with Bruno for 4 nights and it was totally amazing! He took us from the airport, cooked some traditional meals”*

Furthermore, the hosts also gained some cultural learning through similar activities or social practices. For example, some learned about the guests’ culture through food, as guests sometimes cooked traditional food for their host, resulting in the host learning about their culture by spending time with them. In addition, the specific skills of guests were important to co-create value through certain practices, such as performing typical dance rhythms.

- *“She felt very comfortable during the time we lived, and she also cooked recipes from her land that was very good and help me to learn about her culture.”*
- *“They shared with us many interesting things about their culture and their country.”*
- *“We will need the extra samba lessons as the first one didn’t quite stick. Ha-ha not her fault though, she’s a great dancer!”*

##### Engaging Activities

Beyond practices to become acquainted with the destination or contribute to cultural learning, reviews showed that various activities between guests and hosts also contributed to social engagement. As Sevisari and Reichenberger (2020) argued, day-to-day activities provide an opportunity to co-create meaningful encounters between guests and hosts.

For guests, reviews demonstrated how important it was for some of them to spend time with the host doing something that they like to do. In the end, those activities helped to construct a stronger or longer relationship. Some of these activities included: *Having meals with host, practicing a sport, playing games*, or *spending free time together*. The last activity can be explained because hosts were often not in the accommodation because of their jobs, but they tried to use their free time to spend time with guests.

- *“He works a lot but always after job he had time for me. I appreciate everything.”*
- *“Every day we had a nice breakfast together, ones even coffee with salt, hahaha.”*
- *“We did a sport night session of paddling and swimming at the beach. We did a lot of things and I enjoyed everything!”*
- *“They showed us the city, shared their flat and played with us board games. We spend very nice time with them and want to meet again.:)”*

Furthermore, from the host perspective, value emerged through engaging in activities related to the *passions or job* or *leisure activities of guests*, like going to the movies or a spa together.

- *“She is a photographer and took me two photos that I’m sure will be beautiful. We were sharing long conversations about spirituality and about his life.”*
- *I drove her around with my scooter and we went to the cinema and saw Lion King. I was really happy to have her here.”*
- *“I had a wonderful time chatting, laughing, exploring nature, and doing yoga with him. He opens himself.”*

#### Value Co-creation Outcomes

Johnson and Neuhofer (2017) specified that value co-creation outcomes are the result of value co-creation resources and value co-creation practice integration. From the reviews analyzed on Couchsurfing, four major outcomes were identified.

##### Construction of a Friendship Based on Trust

Hosts and guests expressed how the accommodation experience was useful to build a friendship between each other. Opening one’s house to a stranger and the insecurity of traveling alone are aspects that concern users on this platform, thus they want to feel they can trust the other person, and this relationship is achieved merely through interaction. When they spend most of their time together, they often construct a bond that they want to preserve over time.

- *“As a solo female traveler, I felt comfortable and safe staying with him. We did a lot of things and I enjoyed everything! As well as our inspirational conversations and thought exchanges! I won a new friend:)!*
- *“He is incredible! Thanks to Couchsurfing I found my best friend!”*

##### Intention of Repeating the Encounter and Recommendation

Beyond the desire of repeating the experience in the destination, users expressed their wish of repeating the encounter with the same guest or host, even if the destination is different.

- *“I hope to see Oscar again very soon (in Paris maybe) and I highly recommend him”!*
- *“I hope to see you again somewhere in the world”!*
- *“Really hope to see you again soon, perhaps in Berlin!! Be safe and many hugs to you!”*

Almost all analyzed reviews were positive, proving the value extracted by both users. This translated into good recommendations, and also might motivate other users to stay with the same host or accommodate the same guest.

- *“I would definitely recommend staying with her and I hope to see you again soon Barbara:).”*
- *“I really recommend her as a guest is a very nice girl. A hug and good luck on your subsequent trips.”*

##### Experience the Couchsurfing “Spirit”

For most users, the overall combination of resources and social practices resulted in one of the most important values for Couchsurfers, which is the experience itself. Results proved that for this experience to be achieved, integration of operant and operand resources and social practices generated the value they were looking for.

- “*My friend and I had the exact, true couchsurfing experience we were looking for!”*
- *“He is really true traveler, true couchsurfer. If you want to know more about adventures, find him.”*
- *“She embodies the spirit of CS and I’m thankful to know her, Judith:).”*

### Value Co-creation in HomeExchange

Table 2 shows the resources, practices, and outcomes that emerged from the value co-creation process between hosts and guests in HomeExchange. When interpreting the results, it is worth considering the “*modus operandi*” of the platform, where a host might be a guest at the same time, during a simultaneous exchange. In this case, resources, practices, and outcomes might be the same for both types of users, as a user can be a host and a guest at the same time, but for this study, reviews that clearly showed the hosting experience were used.

#### Value Co-creation Resources

##### Resources of Guests

From the analyzed reviews of guests, two major operand resources were found: *Home* and *Places.*

###### Home

Tonner et al. (2016) argued that HomeExchange emerged as a way of maximizing the largest asset of most families, their home. Guests expressed how important it was for them to stay in a kid-friendly house to accommodate their children. Reviews revealed that most users are couples and whole families that travel together to have a family vacation experience. Therefore, when they arrived at the host’s house, they really appreciated finding toys and rooms prepared for kids as expressed in the following quotes:

- *“Our stay at Ernest home was great, his apartment has everything you need and if you have kids, it is perfect as there are plenty of toys.”*
- *“They also had lots of toys, so our kids were never bored. My daughter loved their toys, and the location is very kid friendly.”*

*Home location* was also a characteristic that helped value emerge for guests. Contrary to Couchsurfing, where reviews showed that users do not mind staying in crowded neighborhoods, HomeExchangers preferred to stay in places in quiet areas that could easily access tourist sites, preferably in neighborhoods where locals live and in sites that facilitate rest and relaxation.

- *“She lives in the neighborhood of Gracia in Barcelona, which is the nicest neighborhood to stay in Barcelona for my opinion. Away from the busiest tourist places and surrounded by nice local stores and markets we felt immediately at home.”*

Tonner et al. (2016) also stated that one of the main criteria when selecting a HomeExchange partner is the destination, but apart from that, users also select a home that makes them feel as if they are at home or better. Reviews also revealed that certain amenities in the home of their host helped guests to co-create more value from their experience, like several rooms, a dishwasher, and a washing machine, as well as a terrace, balcony, or a pool.

- *“We spent a lot of time on their amazing balcony, which captures sun almost all day.”*
- *“There were dishes, washing machine, fruits and. to use.”*
- *“The place perfect equipped and there enough space for all, with beautiful terrace and very calm surroundings. And not to forget the pool on the roof!”*

###### Places

As another operand resource, places in the destination also helped to co-create value for guests. HomeExchange users demand places where they can try authentic local culture. Reviews show that stays on this platform tend to be long, as some guests stay in the host’s home for an entire month. This time encourages guests to adapt their daily routines to that of a local resident, which means buying from local shops and dining at local restaurants. Although they also visit tourist places, the focus is on the lifestyle.

- *“It’s absolutely not the part full of tourists, but an area with locals, nice shops and cute restaurants.”*
- *“The apartment was very close to main city attractions and full of small tapas bars and shops mainly for locals which is amazing.”*
- *“The railway station which also functions as a station on two subway lines (L1 and L2) is right around the corner. We navigated the city mostly by foot and by metro. The beautiful beaches of the city are located a pleasant half hour walk away from the apartment.”*

###### Host

For HomeExchange guests, the host is an operant resource that helped value to emerge. To help value co-creation, reviews indicate that guests prefer that hosts possess certain attributes, such as being *informative*, *concerned*, and *helpful.* Reviews showed that hosts are required to be informative by sharing recommendations about local life, things to do, and cultural information.

- *David was very helpful with information about everything concerning Barcelona and places to go to. Thank you, David!*
- *The exchange and communication with Lucas and Maria were easy and they gave us lots of useful information about their neighborhood, and some other places! We can highly recommend the apartment to others!*

Furthermore, characteristics like *helpful* or *friendly* appeared to be required. During their stay, guests like everything to be under control. For example, when an inconvenience arises, they like their hosts to be helpful and problem-solvers. A nice and friendly host also added value, and not merely one person is considered, but the host’s entire family.

- *She crossed the city 3 times to get our invitation letter for the immigration and not only found a cheaper car park place for us but signed the contract in her name!*
- *Jeannie was very friendly and easy going. She even accepted to add a friend of us in the last minute!!!*

**Communication** is another operant resource involved in this platform. Communication is highly important in this kind of exchange. As the stays are long and users are leaving their home in others’ hands, guests value the possibility of communicating with their hosts before the accommodation experience to know all the details, exchange information, or solve doubts during the stay, and even sometimes afterward because they loved the experience and do not want to lose contact. Reviews revealed that, in order for communication to generate value, it must be *clear*, *constant*, and *efficient.*

- *“From the moment we began the conversation of an exchange they responded quickly, and communication was great. They maintained communication during our vacation, and we loved their home and found it a perfect place to relax.”*
- *“The communication was very clear, Iolanda send us a mail with all the info and made sure a friend was there to give us the keys and help us out if necessary.*
- *“We got answers to all our questions, and the communication was easy before and during the exchange. Thanks a lot for this exchange.”*

##### Resources of Hosts

Reviews of hosts expressed two major operant resources in their value co-creation process: *the guest* and *how the guest left the home.*

###### Guest

Reviews revealed that hosts expect some qualities of their guests for value to emerge from the experience, such as being *nice* and *respectful* with *good language skills*, since being able to speak more than one language often facilitates communication.

- *“Nuria speaks a fantastic German as well as Italian, English and some other languages, but we remained mostly with English- She was respectful, and we would have no hesitation in recommending her as a guest.”*

###### How the Guest Left the Home

Some hosts commented on their reviews that finding their home *clean* and *organized* when they returned was a plus in their experience because they did not want to come back from vacation to a messy home. Furthermore, reviews showed that some hosts claimed that their guests left their home *just as they found it*, in perfect condition, which was the case for most guests. In some cases, the hosts expressed that guests left the accommodation in *even better* conditions because the guests did some repairs.

- Our *apartment was in perfect condition upon return; actually, even better because Hilary made a small repair. Thanks for everything, guys!*

#### Value Co-creation Social Practices

##### Practices of Guests

Reviews indicated that three major practices were relevant to placing high value in the accommodation experience of guests: *enjoying the home as their own, exchanging information with host*, and *feeling like a local.*

###### Enjoying the Home as Their Own

Some practices related to the sense of “domesticity” helped to place value in the guests’ experience. The analysis revealed that although guests like to leave the accommodation to visit the city, they appreciate arriving home and enjoying it as if it were their own, thus feeling like another local. To co-create this value, some operand resources are involved, such as the amenities in the house. Unlike other collaborative accommodation platforms, at HomeExchange, guests have access to the entire house and everything in it. This includes outdoor spaces such as swimming pools, terraces, balconies, along with the car, bicycles, or any other means of transport available that the host owns.

- *“We spent our mornings on the terrace and our days exploring the city.”*

Something that is also different from other accommodation platforms is the fact that guests enjoyed not only taking care of the home and spending a lot of time there but also the possibility of taking care of the host’s pets and plants.

- *“We absolutely love staying at your place. The trampoline and the backyard were a big hit for the kids. Kasper enjoyed feeding the cat. I loved the sun and drinking beer in the backyard.”*

###### Exchanging Information With the Host

Reviews also indicated that, for guests, the practice of exchanging information with their host generated added value in their experience. Contrary to commercial tourism, where travelers have most of their trip scheduled and prepared, guests on this platform are usually motivated by the destination and the home, but they do not have a strict plan once they get there. As such, the interactions with their host, the information the host provides about his or her personal life and way of living, and recommendations for things to do help value to emerge.

- *“They gave us amazing with information not just for tourists but also for shops in the vicinity so we could feel as locals. Thanks a lot for sharing with us.”*

###### Feeling Like a Local

From the combination of resources and practices expressed earlier, the third practice that helped to co-create value was feeling like a local. Besides the interaction with the host, who is a local resident and their first contact with the local culture, guests also enjoy practices that make them feel like a member of the local community. Value appeared from activities like eating in local bars and restaurants, shopping in the neighborhood, and visiting nearby villages or towns. These activities were valuable for them because they used the host’s car to go wherever they wanted to enjoy places that were not touristic.

- *“Neighborhood of Gracia is beautiful, very close to main city attractions and full of small tapas bars and shops mainly for locals which is amazing. The neighborhood has many charming shops and restaurants, and even in August it was not overwhelmed with other tourists. You feel a bit like a local staying there.”*

##### Practices of Hosts

When a simultaneous exchange occurs, the hosts become guests at the same time and face the same practices as their guests. When it is a one-way exchange, and the hosts stay in their home sharing time with guests, they have the possibility of getting to know each other better. From the analyzed reviews, two major practices involving the guests emerged to co-create value in the experience of the hosts: *Communicating with guests during the experience* and *doing activities with the guests (before, during, or after).*

###### Communicating With Guests

As Casado-Diaz et al. (2020) described, technology has facilitated this communication, and users on this platform connect not only through the platform but also using other digital channels such as e-mails, WhatsApp messages, videocall apps, etc. Through communication that usually starts before the accommodation and continues during the whole experience, hosts get to know their guests better and vice versa, helping to strengthen the trust in their relationship.

- *“The contact with the host in advance was without problems and during the stay we kept in touch on WhatsApp. She was very easy to deal with throughout the process and while they were here, she sent me some photos of our cat playing with her son which was lovely to see. They were great guests.”*
- *“It was nice to be talk to them and explain through video-call some details about our home and the exchange.”*

###### Doing Activities With the Guests (Before, During, or After)

When the exchange is simultaneous, hosts have the possibility to share time with the guests and do things together that involve certain resources to co-create value. However, even when the exchange is one way, they also have the opportunity to engage in activities together. Reviews showed that some hosts stay in the accommodation to welcome their guests and leave once the guests are settled. In other cases, guests wait for their hosts before leaving the home to say goodbye or in order to have the opportunity to meet each other if they have not before.

- *“We also had the pleasure to stay some hours together, strolling through the old streets of Vienna, which gave us the possibility to get to know each other better. They treated our home very well and left everything in perfect order”*

In some cases, hosts obtained value from receiving gifts from their guests. Kim and Littrell (2001) described the gift-giving practice in the context of tourism, as vacation souvenirs express positive emotions to close people. On HomeExchange, guests added value to the experience of the hosts when they brought them a gift before or after the stay. In addition, the guests’ kids getting along with the hosts’ kids was also valuable for them.

- “…*they left our home so clean and left us some goodies (delicious cookies and a bracelet made for my daughter by her daughter!”*
- *“Our children had an amazing time with Ana’s kid. They played together all the time*…”

#### Value Co-creation Outcomes

Given the model of the platform, many dynamics can appear. These dynamics can be translated into different resources and social practices involving interactions with guests from which certain value co-creation outcomes emerge, such as *desire of repetition*, *users’ recommendation*, and *construction of a long-term relationship*.

##### Desire of Repetition

Contrary to Couchsurfing, HomeExchange users express their desire to repeat the experience in the same destination —Barcelona—and in the same accommodation. When the home has many extra and luxurious amenities, and when interactions with the other user positively contributed to the experience, users tend to want to repeat the experience.

- *“Communication was excellent, and we would like to repeat the experience. Their home was perfect, but they were great too. We loved Barcelona and all you can do there, even if you travel with kids. They were great guests and great hosts too. Thank you!”*

##### Recommendations of Users

Beyond the simple economic benefits, users engage in sharing accommodation practices to access cultural and social capital inherent to these practices (Sevisari and Reichenberger, 2020). Although there is no monetary exchange between users, the platform works with a point system that allows users to earn points when making one-way or simultaneous exchanges. The points users might acquire are also a motivational element, as the more points they obtain, the more exchanges they can do. As good recommendations are necessary to motivate other users to stay or host someone else, they join the HomeExchange community and earn points to do more exchanges. In general, good recommendations prevail in the HomeExchange experience.

- *“Everything was perfect! We would always do it again and we can absolutely recommend it. Thanks for this perfect swap!”*

##### Construction of a Long-Term Relationship

Reviews showed that, contrary to Couchsurfing, stays tend to be longer on this platform, lasting up to a month. The long duration might help to build a more intense relationship between guests and hosts, thus strengthening trust and allowing the relationship to transcend the exchange.

- *“This was more than an exchange of points for housing. This was what it always should be an exchange of beautiful experiences that become a friendship. We felt blessed to get to meet such authentic people! Always open to express themselves to be able to work together on any issue that might arrive. As we said, we will see each other soon. At least for the next few months.*
- “*I made an exchange with Ana and Carlos and their kids and stayed in their apartment for a month*… *We keep in touch and even we have made plans to meet with them soon. Can warmly recommend this family!*

## Discussion and Conclusion

The study of value co-creation on sharing accommodation platforms has received the attention of several scholars (Jiang et al., 2019; Johnson and Neuhofer, 2017; Thaichon et al., 2020). However, most of the research on this topic has focused on the for-profit accommodation platform, Airbnb, and used a guest-centric view. Using the SD logic theoretical framework, this study aimed to explore the resources, practices, and outcomes resulting from the value co-creation process in non-profit sharing-accommodation platforms and how this co-creation process might contribute to the wellbeing of its users.

Regarding the value co-creation resources for guests on non-profit accommodation platforms, operand and operant resources appear to be similar to the resources that co-create value for guests in Airbnb. The *home* and *places* in the destination as physical resources and the *host* as an intangible resource help to co-create value in the accommodation experience of guests. However, the elements that made those resources valuable seem to be different in non-profit platforms, especially in Couchsurfing. As Johnson and Neuhofer (2017) pointed out, for guests of Airbnb in Jamaica, value was co-created through resources resembling conventional hotel amenities, such as internet, hot water, and air conditioning. However, as the results demonstrated, guests on Couchsurfing are not highly concerned about the amenities they might find and do not expect to have the same amenities as in a hotel. It seems their expectations about physical resources are more basic, and they are highly motivated by the operant resources, such as the hosts and their characteristics. Guests on HomeExchange are more motivated by operand resources instead, confirming what Casado-Diaz et al. (2020) explained about HomeExchangers being attracted not only to the destination itself but also by the house amenities, especially in non-traditional destinations. As an extra operant resource, communication with the host played a determinant role for guests on this non-profit platform. This confirms the findings by Andriotis and Agiomirgianakis (2014), who argued that good communication in the decision process makes reciprocal exchanges, such as those in HomeExchange, seem less risky. However, the results of this study also showed that communication in this kind of platform went beyond the decision process of the accommodation, as it was important to co-create value during and after the accommodation experience.

Resources that co-created value for hosts are mostly operant or intangible resources. For both platforms, the guest was transcendental to co-create value in the host experience, but for the hosts on HomeExchange, an additional operant resource came up: *How the guests leave the accommodation.* It confirms what Andriotis and Agiomirgianakis (2014) pointed out: when hosts open up their homes, they have expectations about how their guests should behave. The way the guests treated their house and helped the hosts co-created value during their exchange experience.

When it comes to co-created practices, results showed that interactions between guests and hosts helped construct richer value co-creation outcomes on non-profit accommodation platforms. This idea is in line with the idea exposed by Tussyadiah and Pesonen (2016), who argued that users look for authenticity and social interaction when engaging in sharing-accommodation platforms. Our study confirms that motivations go beyond economic benefits for this kind of platform, which is more present for Airbnb (Tussyadiah and Zach, 2015). *Hosts and guests doing activities together, learning about each other’s culture, discovering the destination together*, and *communicating and exchanging information* were social practices that co-created value for guests and hosts in non-profit platforms. In the absence of the host, that value may be impossible to achieve. This finding supports previous studies such as Schuckert et al. (2018), who argued that Couchsurfing guests normally do not even have a structured plan for their trips, and that one of their motivations is embracing new countries and cultures. Our study shows that the presence of the host during the stay is relevant to achieve cultural learning, as not only do the guests embrace a new culture, but the hosts also learn about the culture and country of their guests.

In their value co-creation analysis of Airbnb in Jamaica, Johnson and Neuhofer (2017) argued that guests create a bond with the environment through local practices, but their results showed limited host involvement in those practices. This might be the main difference between the value co-creation practices in for-profit and non-profit platforms. Some authors have criticized the way guests and hosts interact on Airbnb. Since the users do not have an opportunity to meet each other in some cases (Cockayne, 2016), the host cannot become a resource to co-create value in the accommodation experience of the guests. Casado-Diaz et al. (2020) argued that when people become involved in home-sharing practices, they are looking for a sense of “sociality” and “domesticity,” which is not usual in traditional hospitality. In this respect, our study shows that the host plays a relevant role in non-profit platforms to co-create a sense of sociality and domesticity. This may differ not only from traditional hospitality but also from Airbnb, as this not only creates value for guests but for hosts themselves from their interaction with the guests.

Finally, the integration of the resources and social practices enabled the configuration of value co-creation outcomes in non-profit sharing accommodation platforms. For both platforms, Couchsurfing and HomeExchange, the outcomes are similar, labeled as *the desire to repeat the experience*, *good recommendations*, the *construction of a long-term relationship*, a *friendship based on trust*, and, finally, the *experience* itself on the platform. These results are in line with previous studies, such as Zgolli and Zaiem (2018), who emphasized that a trusting and intimate relationship between guests and hosts can emerge. As our results show, interactions between guests and hosts are vital to generate a relationship based on trust. On Airbnb, hosts and guests interact before the stay to make arrangements; however, the interaction usually stops once the guest arrives at the accommodation. On non-profit platforms, the interaction is constant, which helps to construct a relationship or even a friendship that continues over the years as the reviews demonstrated. As our results show, after the stay, hosts and guests stayed in touch and even repeated the experience over the years. This proves what Forno and Garibaldi (2015) said, that engaging in home-exchanging practices requires a certain level of trust for both the guests and the hosts, which is constructed through communication before, during, and, sometimes, after the exchange. *Good recommendations* are also a valuable co-created outcome. This is similar to Airbnb, where users recommend each other positively, thus motivating other users to participate in sharing accommodation practices. As Johnson and Neuhofer (2017) explained, value does not stop with the individual’s experience but rather extends to involve other participants.

Conversely to Airbnb, on non-profit platforms, users claimed the accommodation was an experience itself. In this regard, the resources and practices for guests and hosts helped to co-create a valuable experience influenced by the way each platform operates. On Couchsurfing, users indicated that they valued the true spirit of the platform, while users on HomeExchange indicated how home swapping was more of an experience than a simple exchange, confirming what Decrop et al. (2018) suggested. Users join Couchsurfing to look for an experience. When people join a sharing economy platform, beyond economic benefits, they are looking for a real sharing experience with a more altruistic perspective (Sevisari and Reichenberger, 2020). In addition, Casado-Diaz et al. (2020) stated that HomeExchange users’ behavior is motivated by the desire to have meaningful interactions with local communities and authentic local experiences.

Tussyadiah (2016) pointed out how literature in consumer behavior indicates that consumers purchase products or services to satisfy their needs. Although basic needs are equally valued in families, social groups, and cultures, conditions that facilitate or undermine wellbeing depend on specific social contexts (Ryan and Deci, 2001). The value co-creation process is different for a young person, someone traveling alone, a traveler with a specific need in his or her trip, or a person staying for one or two days at someone else’s home—generally the type of guest on Couchsurfing—than for couples or entire families traveling with children to spend a week or a month at someone else’s house—generally the type of guest on HomeExchange. The type of users and their accommodation goals are different. These goals influence their needs, which also means that the resources and practices to co-create value and generate wellbeing may also be different.

Users engage with non-profit accommodation platforms to share, but they also expect value in return, which is referred to as a sense of reciprocity (Tussyadiah, 2016). For guests in for-profit platforms, the value outcomes are more related to the destination, physical resources, and interaction with the local culture. However, hosts are limited in the value co-creation process because most play a passive role during their guests’ stay, and their communication and interaction are scarce, thus their contribution to the experience is merely monetary.

On the other hand, value co-creation outcomes in non-profit platforms are more related to social practices and the interaction between guests and hosts. Results have shown the construction of meaningful relationships between both users, which help value to emerge from their accommodation experience and to contribute to their wellbeing. As Ryan and Deci (2001) argued, when people feel understood, engaged in meaningful dialog, or had fun with others, they are able to experience wellbeing. Therefore, the social dimension of wellbeing is achieved through social practices and interaction between the hosts and guests.

Results also show that not only do the social practices contribute to the wellbeing of users but also the resources. When HomeExchange users, for example, exchange their home, they are also opening up the opportunity for themselves to stay at another home around the world to have a vacation and time to relax as a family (Mccrea et al., 2016), which might increase their wellbeing.

Regarding theoretic contributions, this paper aims to fill the literature gap regarding the value co-creation process in non-profit sharing-accommodation platforms. Scarce literature refer to the dynamics that might be happening within non-profit accommodation platforms (Medina-Hernandez et al., 2020). By means of the SD logic approach, this study analyzes value co-creation resources, practices, and outcomes in different models of non-profit accommodation platforms and links them with their potential contribution to the wellbeing of their users. This study also highlights the potential role of both users, guests, and hosts in the value co-creation process and underlies how capitalist practices in for-profit platforms may undermine the value that social and sharing practices can contribute to the wellbeing of individuals.

As far as practical implications are concerned, this study provides practical implications for sharing accommodation platform owners and practitioners. Findings show the importance of interactions between guests and hosts as a value co-creation practice for the accommodation experience of users and their necessity of involvement in authentic experiences. In this regard, destination marketing organizations (DMOs) could incentivize local businesses to collaborate with sharing accommodation platforms, offering discounts or special prices for hosts and guests to attend events or visit places together. This may boost their sharing experience, contribute to local and small business revenues, and add new resources and practices to the guest–host value co-creation process. Non-profit accommodation platform owners could also provide information on the platform about local plans and events at the destination and offer extra points for users who attend these together. In addition, practitioners could design and implement short tours for Couchsurfing guests and hosts, given the nature of their brief stays and the importance of resources as “places” and practices such as “touring and discovering the destination together” for users in that platform. This could not only help co-create more valuable experiences but also improve the tourist destination image. Resources and practices allow users to extract value in-context and in-use (Vargo and Lusch, 2004; Johnson and Neuhofer, 2017). Thus, practitioners and policymakers can design strategies using resources and practices from which guests and hosts could extract value and contribute to the wellbeing of both as well as the community’s wellbeing. An example is discounts for children’s activities, as results show that most HomeExchange users travel with their families, or city tours adapted to less frequently visited neighborhoods and more congested areas, given the importance HomeExchange users place on having local experiences. Airbnb reviews from Barcelona were also analyzed for this study but not included in the results section because the findings were similar to previous studies, and our focus was on analyzing value co-creation in non-profit accommodation platforms. Thus, comparisons between for-profit and non-profit platforms can still be carried out, and the main difference between both models concerns the absence of the host.

As far as limitations and further research, since this study is geographically limited to the city of Barcelona, the resources and practices are related to that destination. Future research of value co-creation in non-profit platforms could analyze whether users extract similar value from different or equal practices and resources in other destinations. For this study, only reviews in the English language were considered. Further research should consider expanding the language spectrum. Also, data were extracted in a pre-COVID-19 scenario, which could lead to different results at the time this study was published in 2021. The coding structure adopted in the qualitative analysis may involve a certain degree of subjectivity. Future lines of investigation could go further than the codification raised in this study, which was based on Johnson and Neuhofer (2017), to see whether additional elements influence the value co-creation extraction.

## Data Availability Statement

The data analyzed in this study is subject to the following licenses/restrictions: Data analyzed can be seen just if a personal account in each platform is created. Requests to access these datasets should be directed to www.couchsurfing.com/es/ and www.homeexchange.com/es.

## Author Contributions

VM-H, BF-R, and EM-R contributed to the conception and design of the study. VM-H organized the data collection, performed the qualitative analysis, wrote the first draft of the manuscript, discussed, and interpreted the results. BF-R and EM-R contributed to manuscript revision and read and approved the submitted version. All authors contributed to the article and approved the submitted version.

## Conflict of Interest

The authors declare that the research was conducted in the absence of any commercial or financial relationships that could be construed as a potential conflict of interest.

## Publisher’s Note

All claims expressed in this article are solely those of the authors and do not necessarily represent those of their affiliated organizations, or those of the publisher, the editors and the reviewers. Any product that may be evaluated in this article, or claim that may be made by its manufacturer, is not guaranteed or endorsed by the publisher.
